# Esports Players Are Less Extroverted and Conscientious than Athletes

**DOI:** 10.1089/cyber.2022.0067

**Published:** 2023-01-17

**Authors:** Maciej Behnke, Michal M. Stefanczyk, Grzegorz Żurek, Piotr Sorokowski

**Affiliations:** ^1^ Department of Psychology and Cognitive Science, Adam Mickiewicz University, Poznań, Poland.; ^2^ Institute of Psychology, Faculty of Historical and Pedagogical Sciences, University of Wrocław, Wrocław, Poland.; ^3^ Faculty of Physical Education, Wroclaw University of Health and Sport Sciences, Wroclaw, Poland.

**Keywords:** esports, gamers, athletes, personality, extroversion, conscientiousness

## Abstract

The worldwide status of esports as a sporting phenomenon has been developed in the past decade. However, as the esports industry has grown, it has remained an understudied scientific field. Esports is often contrasted with traditional sports regarding various aspects, including lack of physical activity and the online nature of social interactions. However, little is known whether individuals competing in esports—esports players—differ from individuals competing in traditional sports—athletes. To address this question, we examined the personality characteristics of both types of performers. We collected cross-sectional data on esports players' (*n* = 416) and athletes' *(n* = 452) personalities and performance characteristics. We found that esports players were less extroverted and conscientious than athletes. Furthermore, greater sports and esports experience was positively related to being more extroverted. Our findings contribute to the literature by documenting the preferences for competitive activities based on individuals' personality characteristics. We suggest that esports (rather than sports) might be a more suitable form of competition for less extroverted and conscientious individuals.

## Introduction

Esports (electronics sports) is the fastest-growing area in sports, in which individuals compete using video games. Esports recently met the formal definition of sports^1^ because it is an institutionalized and international form of competition where success depends on precise movements and high cognitive skills.^2^ Esports players are people playing esports games that regularly engage in deliberate practice (i.e., gaming and nongaming activities focused on enhancing specific esports skills). The esports industry is growing extraordinarily fast, with more than 3 billion video gamers worldwide (in comparison with less than 2 billion in 2015)^3,4^ and 100 million esports players.^5,6^

Although esports status as a worldwide phenomenon is growing, esports remains an understudied scientific field. Many recent reviews point to the internal inconsistency of definitions and viewpoints.^2,7,8^ The same applies to the psychological perspective of professional esports players and the factors that lead to successful esports performance.^9–11^ Thus, considering the growing numbers of active esports players and the esports industry, we aimed to examine esports players' personalities.

Personality—a set of consistent patterns of feelings, thinking, and behaviours^12^—provides basic information about an individual and is one of the most studied human characteristics. Researching personality in distinctive groups (such as esports players) might help understand the mechanisms behind professional development and provide targeted help. In esports, taking care of players' mental and physical health is crucial, considering how demanding professional esports is.^13,14^ For instance, esports players tend to practice 12–14 hours a day, 6 days a week, and live in shared homes with other players.^13,15,16^ The high demands may lead to psychological burnouts, quitting a career by mid-20s, and health problems, including sleep disturbances^17^ and musculoskeletal pain^18^—issues that traditional sports professionals share, too.^19^

However, unlike esports players (except for a study on differences among League of Legends players),^20^ athletes as members of a distinct social/vocational group are relatively well studied.^21–23^ Athletes, compared with nonathletes, are more conscientious,^24^ extroverted,^25^ open to experience,^26^ and less neurotic.^27^ Furthermore, there are differences among athletes, namely team sports athletes are more extroverted and less conscientious than individual sports athletes.^28–31^ Athletes practicing extreme sports are more extroverted and less conscientious than athletes practicing low-risk sports.^32–35^ Combat athletes are less neurotic than team sport athletes,^31^ and even within team sports, athletes differ in neuroticism, extroversion, agreeableness, and conscientiousness.^36^ Top-level athletes are more conscientious and less neurotic than lower-level athletes.^30,36–38^ Studies examining the differences between esports players and nongamers found that esports players were less neurotic and conscientious than nongamers.^39,40^

The individuals' personality characteristics might result in preferences for specific competitive activities that, in turn, ultimately distinguish a specific group of athletes. Thus, in this study, we focus on whether individuals competing in esports—esports players—differ from individuals competing in traditional sports—athletes. We ran a cross-sectional study with two groups of participants: esports players and athletes, to explore possible personality (in)differences. We hypothesized that esports players would be less extroverted (H1), less conscientious (H2), and more neurotic (H3).

We developed our hypothesis based on three premises differentiating esports from traditional sports. First, esports involves much less physical social interaction, so that it might be best suited to less extroverted individuals. Second, esports requires spending many hours in sitting positions. Studies found that individuals spending more leisure time sitting tend to be less conscientious, extroverted, open to experience, and more neurotic.^41^ Third, esports require less physical activity than traditional sports. Meta-analyses show that more extroverted and conscientious but less neurotic individuals are also more physically active.^42,43^

## Materials and Methods

### Participants

Participants were esports players and athletes (*N* = 868) (for details, see Table 1). Using a-priori Sample Size Calculator for Multivariate Regression Model,^44^ we determined that at least 827 participants were needed to detect small effect sizes with the power of at least 0.90 in our model. We recruited esports players and athletes through a Facebook advertisement targeted at esports\sports enthusiasts. The Research Ethics Committee of the Institute of Psychology at the University of Wrocław approved the study (approval no. 2022/LNCGQ). Participation in the study was voluntary, and each participant provided informed consent. The study was in accordance with The Code of Ethics of the World Medical Association (Declaration of Helsinki).

**Table 1.** Participants Characteristics

Participants	Esports players (*n* = 416)		Athletes (*n* = 452)	
Age, mean, SD	23.13, 5.01		27.14, 8.72	
Gender, *n* (percent)				
Woman	69 (16.6)		122 (27.0)	
Man	347 (83.4)		330 (73.0)	
Performance level, *n* (percent)				
Recreational	237 (57.0)		79 (17.5)	
Local	107 (25.7)		129 (28.5)	
National	46 (11.1)		177 (39.2)	
International	26 (6.3)		67 (14.8)	
Professional level, *n* (percent)				
No-income activity	356 (85.6)		334 (73.9)	
Part-time job	56 (13.5)		96 (21.2)	
Full-time job	4 (1.0)		22 (4.9)	
Participation in sport/esport (years), mean, SD	6.48, 3.96		9.30, 5.30	
Daily training (hours), mean, SD	3.61, 2.06		2.18, 1.32	
Primary activities, *n* (percent)	League of legends	111 (26.7)	Running	86 (19.0)
	Counter strike: global offensive	94 (22.6)	Soccer	61 (13.5)
	World of tanks	47 (11.4)	CrossFit	35 (7.7)
	Dota 2	34 (8.2)	Triathlon	35 (7.7)
	FIFA	33 (7.9)	Track and field	33 (7.3)

SD, standard deviation.

### Measures

#### Personality

We assessed personality with the Ten Item Personality Inventory (TIPI-PL) personality questionnaire^45^ (Polish adaptation).^46^ The TIPI-PL is based on the Big Five personality model (Extroversion, Neuroticism, Openness to Experience, Conscientiousness, and Agreeableness) and consists of 10 items, two for each dimension. Participants rated how much they thought each scale applied to them on a 7-point scale ranging from 1 (*definitely disagree*) to 7 (*definitely agree*). As recommended for 2-item scales,^47^ the Spearman–Brown rho showed acceptable reliability^48,49^ for Extroversion (ρ = 0.65), Neuroticism (ρ = 0.72), Conscientiousness (ρ = 0.71), and low reliability for Agreeableness (ρ = 0.55) and Openness to experience (ρ = 0.26).

The items within the dimensions show similar correlations to the questionnaire validation study^46^ (Supplementary Data). The reliability of some of the TIPI scales in our study was low. Thus, we interpreted only the results for extroversion, neuroticism, and conscientiousness scales, and we present data and results for agreeableness and openness to experience scales for exploratory purposes. We used a short questionnaire so that participants—including professional athletes—could complete it, which would be difficult with a long scale.

#### Performance

Participants reported their primary sport/esport played, performance level (the highest level of competition: recreational, local, national, and international), professional level (sport/esport as full-time job, part-time job, and no-income activity), experience (duration of participation in sport/esport; in years), duration of daily training (in hours for a typical day).

### Statistical analysis

First, we recoded the reverse items from the TIPI questionnaire and removed outliers above z-scores higher than 3.29^50^ (Supplementary Data). Next, we calculated pairwise comparisons for the differences in personality dimensions between esports players and athletes reported as effect sizes (Cohen's *d*) with confidence intervals (95% CIs). The differences between groups would be supported if CIs for Cohen's *d* would not include zero.^50^

Finally, we ran a multivariate regression model using Mplus 8.0. In the model, we regressed five personality dimensions (sum of two items) on sports group (dummy coded athletes vs. esports players), duration of participation in sport/esport, daily training duration, age, gender (dummy coded man vs. woman), competition level (dummy coded recreational vs. local or national or international) and professional level (dummy coded no-income activity vs. part-time job or full-time job). Our hypotheses would be supported if the CIs for regression coefficients will not include zero.^50^ We calculated the same number of parameters a model can estimate, so we expected the model to have perfect fit indices.^51^ We present the data and the code used for analyses in Supplementary Materials (BehnkeMplusData2 and BehnkeMplusSyntax).

## Results

### Univariate differences in personality

Table 2 presents descriptive statistics for esports players and athletes. We found that esports players are less extroverted and conscientious but more neurotic than athletes. The size of effect sizes should be interpreted as medium sized.

**Table 2.** Descriptive Characteristics and Differences in Personality Dimensions Between Esports Players and Athletes

Variable	Esports players		Athletes		d	Difference
	Mean	SD	Mean	SD		95% CI
Extroversion	9.71	3.49	10.95	2.91	−0.39	−0.52 to −0.25
Conscientiousness	9.61	3.19	11.38	2.57	−0.62	−0.75 to −0.48
Neuroticism	7.44	3.63	6.40	3.35	0.30	0.17 to 0.43
Agreeableness^a^	5.84	2.74	5.04	2.41	0.31	0.18 to 0.44
Openness to experience^a^	9.41	2.61	9.73	2.45	−0.13	−0.26 to 0.00

^a^ Owing to the low reliability of the subscales, the results are presented for exploratory purposes and should be interpreted cautiously. Esports players *n* = 416 and athletes *n* = 452.

CI, confidence intervals; *d*, Cohens' *d*.

### Multivariate regression model

The model had perfect fit, for baseline model chi-squared (45) = 719.02, *p* < 0.01, root-mean-squared error of approximation = 0.00, comparative fit index = 1.00, and standardized root mean of the residual = 0.00. Full results are presented in Tables 3 and 4. Similar to univariate analysis, we found that esports players, compared with athletes, were less extroverted and conscientious (Table 4). Individuals with more experience in sports and esports were more extroverted. Women were more conscientious and neurotic than men in our sample. Older individuals were less neurotic.

**Table 3.** Correlations Between the Measures Included in the Multivariate Regression Model

	1	2	3	4	5	6	7	8	9	10	11
1. Group	—										
2. Extroversion	−0.19^**^	—									
3. Agreeableness^a^	0.15^**^	−0.18^**^	—								
4. Conscientiousness	−0.30^**^	0.23^**^	−0.25^**^	—							
5. Neuroticism	0.15^**^	−0.44^**^	0.20^**^	−0.25^**^	—						
6. Openness to experience^a^	−0.07	0.33^**^	−0.11^**^	0.05	−0.14^**^	—					
7. Participation in sport/esport	−0.29^**^	0.18^**^	−0.04	0.15^**^	−0.17^**^	0.06	—				
8. Daily training	0.39^**^	−0.06	0.14^**^	−0.08^*^	0.09^**^	−0.05	−0.03	—			
9. Competition level	−0.42^**^	0.13^**^	−0.07^*^	0.17^**^	−0.12^**^	0.05	0.31^**^	0.03	—		
10. Professional level	−0.16^**^	0.09^**^	−0.03	0.06	−0.03	0.03	0.24^**^	0.21^**^	0.43^**^	—	
11. Age	−0.27^**^	0.11^**^	−0.10^**^	0.14^**^	−0.23^**^	0.06	0.31^**^	−0.23^**^	0.06	−0.12^**^	—
12. Gender	−0.13^**^	0.01	−0.09^**^	0.15^**^	0.21^**^	0.07^*^	−0.02	−0.08^*^	−0.04	−0.03	0.11^**^

*Note*: Group coded as athletes = 0 and esports players = 1; competition level coded as recreational athletes/esports players = 0 and local, national, or international = 1; professionalization level coded as no-income activity = 0 and part-time job or full-time job = 1; gender coded as men = 0 and women = 1.

^a^ Owing to low reliability of the subscales, the results are presented for exploratory purposes and should be interpreted cautiously.

^*^ *p* < 0.05, ^**^p < 0.01.

**Table 4.** Full Results for Multivariate Regression Model

Outcome	Predictors	Estimate	95% CI
Extroversion	Group	−0.112	−0.195 to −0.030
	Experience	0.110	0.038 to 0.182
	Training time	−0.009	−0.083 to 0.065
	Age	0.045	−0.028 to 0.117
	Gender	0.001	−0.065 to 0.067
	Professional level	0.036	−0.036 to 0.109
	Performance level	0.063	−0.013 to 0.139
Conscientiousness	Group	−0.235	−0.314 to −0.156
	Experience	0.057	−0.014 to 0.127
	Training time	0.032	−0.040 to 0.104
	Age	0.052	−0.018 to 0.122
	Gender	0.127	0.063 to 0.190
	Professional level	0.000	−0.071 to 0.070
	Performance level	0.063	−0.011 to 0.137
Neuroticism	Group	0.054	−0.025 to 0.134
	Experience	−0.067	−0.137 to 0.002
	Training time	0.044	−0.028 to 0.115
	Age	−0.207	−0.276 to −0.139
	Gender	0.233	0.171 to 0.295
	Professional level	−0.015	−0.085 to 0.055
	Performance level	−0.071	−0.144 to 0.002
Agreeableness^a^	Group	0.071	−0.012 to 0.154
	Experience	0.032	−0.040 to 0.105
	Training time	0.105	0.031 to 0.180
	Age	−0.068	−0.141 to 0.004
	Gender	−0.070	−0.137 to −0.004
	Professional level	−0.080	−0.153 to −0.007
	Performance level	−0.032	−0.108 to 0.045
Openness to experience^a^	Group	−0.011	−0.096 to 0.073
	Experience	0.042	−0.032 to 0.116
	Training time	−0.035	−0.111 to 0.041
	Age	0.027	−0.047 to 0.101
	Gender	0.066	−0.002 to 0.133
	Professional level	0.010	−0.064 to 0.084
	Performance level	0.027	−0.051 to 0.105

*Note*: Group coded as athletes = 0 and esports players = 1; gender coded as men = 0 and women = 1, competition level coded as recreational athletes/esports players = 0 and local, national, or international = 1; professional level coded as no-income activity = 0 and part-time job or full-time job = 1; gender coded as men = 0 and women = 1.

^a^ Owing to low reliability of the subscales, the results are presented for exploratory purposes and should be interpreted cautiously.

## Discussion

We examined the differences in personality characteristics between esports players and athletes. Using univariate and multivariate approaches, we found that esports players were less extroverted and conscientious than athletes. The differences in extroversion might result from the less physical nature of social interaction in esports compared with sports. Thus, individuals who prefer indirect interaction with others might be more willing to engage in esports competition. Furthermore, more extroverted people tend to be more physically active^42,43^ and spend less leisure time sitting.^41^

Thus, individuals who prefer to be physically active might be more willing to participate in traditional sports competitions. Although physical activity level might be the most apparent difference between esports and traditional sports, it does not necessarily mean that esports players are physically inactive. Esports players report higher physical activity levels than the World Health Organization recommendations^52^ (which increases as players become more professional).^53,54^ Physical activity prepares their bodies for multihour gaming sessions in the seated position, maintaining health and fastening training recovery.^55,56^

The differences in conscientiousness might result from engaging in a specific form of activity.^23^ Esports, compared with sports, is still a less structured and organized form of activity, especially at the early stages of the esports career. Traditional sports created the net of clubs and places where young athletes can develop their skills under the supervision of well-educated coaches. This net is just developing in esports, so the nature of the esports competition is also less time-restricted. Esports players train their skills independently and do not have to follow the clubs' workout schedules. These environmental differences may shape the individuals' conscientiousness differently.

Our findings contribute to the literature by documenting the preferences for doing competitive activities based on individuals' personality characteristics. The ability to predict individuals' preferences might have practical implications. Parents and coaches may guide a young performer to select or reject appropriate activities or even specific roles/positions within sports or esports. It would not be practical to develop programs and interventions to suppress or express any personality characteristics, as they are consistent and enduring^12^—it would be better to direct individuals toward activities they are best suited for.

Contrary to our hypothesis, when controlling for the competition and professional level of athletes and esports players, we did not find differences in neuroticism (the difference observed with the univariate approach). Our findings show the advantage of using multivariate statistical models when studying personality. In our model, we also tested individual differences (e.g., experience and age) as predictors of personality. We found that the longer the sport or esports experience, the higher the extroversion levels.^57^ Like in the general population,^58,59^ women in the performance domain were more neurotic than men, and older esports players and athletes were less neurotic. We also found that women were more conscientious than men.

### Limitations and future research

First, our research design was cross-sectional. Longitudinal designs would provide direct evidence of whether specific individuals engage in the different forms of activities or whether the activities, to some extent, impact individuals' personalities. Further studies might test it by starting the studies with minors. Such studies would also be crucial in identifying processes that could prevent problematic gaming. Second, we used a brief questionnaire to assess personality. Although the scale is widely used in personality research, its structure does not allow for building more parsimonious analytical models with latent factors. Furthermore, as in the original version of the TIPI^45^ and its Polish adaptation,^46^ we found medium-sized correlations between the items accounting for the same personality trait.

Therefore, results related to openness to experience and agreeableness, which show the smallest correlation and low reliability, should be interpreted cautiously. Third, although esports is a growing global phenomenon, we focused on Polish samples. Further studies might test whether the same difference appears in more and less industrialized countries and cultures. Finally, we treated the esports players and athletes as homogeneous groups. Studies in traditional sports indicate differences between sports disciplines.^36–38^ Thus, future research may focus on identifying personality characteristics unique to specific esports genres (e.g., first-person shooter games, massively multiplayer online role-playing games), specific esports games (e.g., Fortnite and League of Legends)^20^ or competition and professional levels (e.g., local vs. international esport players).

## Conclusion

We report novel findings on how esports players differ from athletes. These findings are essential, given that esports is often presented as displacing traditional sports. We emphasize that esports is not a substitute for traditional sports, but esports might be a more suitable form of competition for less extroverted and conscientious individuals.

## Supplementary Material

Supplemental data
