# Supplementary material for: Esports Players Are Less Extroverted and Conscientious than Athletes
Source: Cyberpsychol Behav Soc Netw. 2023 Jan 17;26(1):50–6. doi: 10.1089/cyber.2022.0067 (PMC9885544; doi:10.1089/cyber.2022.0067)
Supplement: Supplemental data [file Supp_Data.zip › Supplementary Materials.docx]

**Supplementary Materials**

**S2. MATERIALS AND METHODS**

**S2.1 Participants**

From the initial sample (*N* = 927), we removed 59 outliers or people who did not provide full data (10 for not reporting their sex, 11 for participation in sport/esport duration, 12 for the duration of daily training, 26 for not reporting their duration of daily training, seven for duration of daily training participation in sport/esport duration).

**S2.2 Measures**

*S2.2.1 Personality*

The items within the dimensions show similar correlations to the questionnaire validation study^45^, namely, Extraversion, *r* = -.48, *p* < .001, Neuroticism, *r* = -.57, *p* < .001, Openness to experience, *r* = .15, *p* < .001, Conscientiousness, *r* = -.55, *p* < .001, and Agreeableness *r* = -.38, *p* < .001. We note that the internal consistency of some of the TIPI's scales in our study was low, yet this is considered typical, given the length of this questionnaire^46^, and is similar to the previous studies^s1^. We used a short questionnaire so that many participants - including professional athletes - could complete it, which would be more difficult with a longer scale.

| **Table S1**  *Correlations Between Items Within Personality Dimensions* | | | |
| --- | --- | --- | --- |
|  | Our Study | Polish adaptation  (Sorokowska et al., 2014) | Original Study (Gosling et al., 2003 |
| Extraversion | -.48 | -.53 | -.56 |
| Agreeableness | -.38 | -.43 | -.36 |
| Conscientiousness | -.55 | -.61 | -.42 |
| Neuroticism | -.57 | -.57 | -.61 |
| Openness to experience | .15 | -.28 | -.28 |

**S2.3 Statistical analysis**

*S2.3.1 Exploratory Analysis*

We explored the differences between esports players and athletes with multivariate ANOVA.

**S3. RESULTS**

We found a statistically significant difference in demographics between gamers and esports players, *F* (6, 861) = 90.51, *p* < .001; Wilk's Λ = 0.61, partial η2 = .39. We found that gamers were younger, spent more time on daily training, but had lower experience than athletes (Table S2). Furthermore, the sample of gamers was more dominated by men and recreational and non-paid participants than the sample of athletes (Table S2).

| **Table S2**  *Demographic differences between esports players and athletes* | | | | | | | | |
| --- | --- | --- | --- | --- | --- | --- | --- | --- |
|  | Athletes | | | Esports Players | | | *F* | η2 |
|  | *M* | *SD* | *N* | *M* | *SD* | *N* |  |  |
| Experience | 9.31 | 5.31 | 452 | 6.49 | 3.97 | 416 | 77.52^***^ | 0.08 |
| Daily training | 2.18 | 1.32 | 452 | 3.62 | 2.07 | 416 | 150.83^***^ | 0.15 |
| Age | 27.15 | 8.72 | 452 | 23.13 | 5.01 | 416 | 67.74^***^ | 0.07 |
| Sex | 0.27 | 0.44 | 452 | 0.17 | 0.37 | 416 | 13.85^***^ | 0.02 |
| Professionalization level | 0.26 | 0.44 | 452 | 0.14 | 0.35 | 416 | 18.48^***^ | 0.02 |
| Competition level | 0.83 | 0.38 | 452 | 0.43 | 0.50 | 416 | 175.03^***^ | 0.17 |
| *Note.* For all variables *df* =1,868. Sex = Percentage of women, Competition level = percentage of non-recreational athletes/esports players, Professionalization level = percentage of paid athletes. Experience = Participation in sport/esport (years), Daily training is reported in hours. | | | | | | | | |

**REFERENCES**

^s1^Zielińska A, Lebuda I, Ivcevic Z, et al. How adolescents develop and implement their ideas? On self-regulation of creative action. Think Skills Creat 2022;43:100998. doi: 10.1016/j.tsc.2022.100998
